# New tools for investigating astrocyte-to-neuron communication

**DOI:** 10.3389/fncel.2013.00193

**Published:** 2013-10-29

**Authors:** Dongdong Li, Cendra Agulhon, Elke Schmidt, Martin Oheim, Nicole Ropert

**Affiliations:** ^1^Biophysics of Gliotransmitter Release Team, Laboratory of Neurophysiology and New Microscopies, INSERM U603, CNRS UMR 8154, University Paris DescartesParis, France; ^2^Glia-Glia and Glia-Neuron Interactions in Neurophysiopathology Team, Laboratory of Neurophysiology and New Microscopies, INSERM U603, CNRS UMR 8154, University Paris DescartesParis, France

**Keywords:** photoactivation, pharmacogenetics, optogenetics, gliotransmission, GCaMP, LiGluR, CatCh, ChR2

## Abstract

Gray matter protoplasmic astrocytes extend very thin processes and establish close contacts with synapses. It has been suggested that the release of neuroactive gliotransmitters at the tripartite synapse contributes to information processing. However, the concept of calcium (Ca^2+^)-dependent gliotransmitter release from astrocytes, and the release mechanisms are being debated. Studying astrocytes in their natural environment is challenging because: (i) astrocytes are electrically silent; (ii) astrocytes and neurons express an overlapping repertoire of transmembrane receptors; (iii) the size of astrocyte processes in contact with synapses are below the resolution of confocal and two-photon microscopes (iv) bulk-loading techniques using fluorescent Ca^2+^ indicators lack cellular specificity. In this review, we will discuss some limitations of conventional methodologies and highlight the interest of novel tools and approaches for studying gliotransmission. Genetically encoded Ca^2+^ indicators (GECIs), light-gated channels, and exogenous receptors are being developed to selectively read out and stimulate astrocyte activity. Our review discusses emerging perspectives on: (i) the complexity of astrocyte Ca^2+^ signaling revealed by GECIs; (ii) new pharmacogenetic and optogenetic approaches to activate specific Ca^2+^ signaling pathways in astrocytes; (iii) classical and new techniques to monitor vesicle fusion in cultured astrocytes; (iv) possible strategies to express specifically reporter genes in astrocytes.

## Introduction

The concept of gliotransmission at the tripartite synapse developed more than 10 years ago (Araque et al., 1999; Perea and Araque, 2010) is very attractive: it suggests that cerebral gray matter protoplasmic astrocytes are not only supportive cells with homeostatic functions, but that they also play a role in information processing by responding to neuronal synaptic activity with Ca^2+^ elevations that induce the subsequent release of gliotransmitters and modulate neuronal excitability and synaptic plasticity [reviewed in (Angulo et al., 2008; Bergersen and Gundersen, 2009; Cali et al., 2009; Perea et al., 2009; Santello and Volterra, 2009; Halassa and Haydon, 2010; Perea and Araque, 2010; Parpura et al., 2011; Gucek et al., 2012; Zorec et al., 2012)]. However, the concept of gliotransmission is debated. First, the ability of astrocytes to release neuroactive compounds in a Ca^2+^-dependent manner has been questioned (Agulhon et al., 2008; Fiacco et al., 2009). Second, there is no consensus concerning the mechanisms of gliotransmitter release. In fact, studying astrocytes *in situ* is very challenging and an agreement is emerging that new methods are needed to selectively activate (Fiacco et al., 2009; Hamilton and Attwell, 2010) and read out Ca^2+^ signals in astrocytes *in situ* (Shigetomi et al., 2013b).

By gliotransmission, we mean Ca^2+^-dependant release of fast-acting neuroactive compounds, the gliotransmitters. Astrocytes can also release other molecules acting not only on neighboring neurons but also on nearby glial cells such as microglia, NG2 cells, and on cellular constituents of the blood brain barrier. Our review will focus on the fast acting gliotransmitter candidates, glutamate mostly, as well as D-serine, ATP, and GABA. Several pathways have been suggested for glutamate release: exocytosis, hemichannels, sodium-dependent transporters, volume-regulated anion channels, purine P2X7 receptor channel [reviewed in (Hamilton and Attwell, 2010)], and more recently the bestrophin 1 (Best1) chloride channel and the two-pore domain potassium TREK1 channel (Woo et al., 2012). The release of glutamate by Ca^2+^-regulated vesicular fusion is considered as an important pathway for gliotransmitter release because, by analogy with neuronal exocytosis, it appears to be the most suitable pathway for rapid information processing by astrocytes. Experimental evidence in favor of glutamate exocytosis has been provided using (i) dihydroxyphenylglycine (DHPG), mechanical stimulation, inositol triphosphate (IP_3_) and Ca^2+^ uncaging to activate astrocytes; (ii) Ca^2+^ buffering with BAPTA, VAMP2/3 cleaving with tetanus toxin (TeNT) or botulinum toxin (BoNT), and generating a dominant negative SNARE (dnSNARE)-expressing mouse line to inactivate vesicular release in astrocytes; (iii) fluoroacetate to inactivate astrocyte metabolism [reviewed in Bergersen and Gundersen, 2009; Cali et al., 2009; Gucek et al., 2012; Zorec et al., 2012], but contrasts with the relative absence on electron micrographs of small vesicles in astrocyte processes, when compared to the neuronal presynaptic terminal.

In this review, we discuss several new genetically encoded tools to read out astrocytic Ca^2+^ activity, to activate Ca^2+^ signals in astrocytes, and to monitor gliotransmitter release from astrocytes. Optical methods for astrocyte photoactivation and imaging, and strategies to selectively target the genes in astrocytes in their native environment are also reviewed.

## Imaging astrocyte activity

### Organic vs. genetically encoded Ca^2+^ indicators

Protoplasmic astrocytes are electrically silent. However, they may be considered as excitable cells in the sense that they show Ca^2+^ signals, both spontaneously and in response to neuronal activity. In spite of the evidence suggesting that Ca^2+^ signals are necessary and sufficient to induce gliotransmitter release, many questions remain, concerning both the role and sources of Ca^2+^ signals in astrocytes (Agulhon et al., 2008; Fiacco et al., 2009; Parpura et al., 2011). One limiting factor to study Ca^2+^ signaling has been methodological. So far, most studies in acute brain slices and *in vivo* have been based on bulk-loaded membrane-permeable chemical Ca^2+^ indicators. There exist many organic Ca^2+^ indicators with different spectral properties and affinity for Ca^2+^ which can monitor either Ca^2+^-related fluorescence changes or, for the ratiometric probes, can be calibrated to provide absolute Ca^2+^ concentration [reviewed in (Paredes et al., 2008)]. The membrane-permeable acetoxymethyl (AM) esters, Fluo4-AM and Oregon Green BAPTA1-AM (OGB1-AM), the most popular dyes used to image Ca^2+^ activity in populations of astrocytes, allow imaging the somatic region and the larger proximal processes but leave the very thin distant processes that participate to the tripartite synapse unsampled (Reeves et al., 2011). At the laser powers, dye concentrations and integration times typically used and with the spatial resolution available, fluorescence changes in fine processes are generally not resolved (see below for a detailed discussion).

Another limiting factor has been the lack of specificity of these membrane-permeable Ca^2+^ indicators, which label both neurons and astrocytes (Garaschuk et al., 2006) with cell-type preferences, depending on the indicator, the protocol of application, and the age of the animal. Therefore, sulforhodamine 101 (SR101) that is specifically taken up by astrocytes has been generally used as a secondary fluorescent marker for astrocyte identification. The deep-red emission of SR101 can be detected with negligible spectral overlap with GFP or green fluorescein-based membrane-permeable Ca^2+^ indicators (Nimmerjahn et al., 2004). However, SR101 uptake is age-dependent (Kafitz et al., 2008) and it does not work in all brain regions (Schnell et al., 2012). Also, at concentrations needed for astrocyte labeling, SR101 leads to increases of neuronal excitability and long-term potentiation (Kang et al., 2010; Garaschuk, 2013), and thus might affect functional studies. As an alternative, transgenic (Tg) mouse lines (Nolte et al., 2001; Vives et al., 2003; Heintz, 2004; Zuo et al., 2004; Regan et al., 2007) expressing a green/yellow fluorescent protein (GFP/YFP) or *Discosoma* red protein (DsRed) under astrocyte-specific promoters (GFAP, S100β, GLT-1, ALDH1L1) can be used to identify astrocytes.

In this context, the recent genetically encoded Ca^2+^ indicators (GECI) provide a new alternative for non-invasive imaging of Ca^2+^ activity *in vivo* and in brain slices [reviewed in (Knopfel, 2012; Looger and Griesbeck, 2012)]. Their level of expression can be stable for months in the absence of apparent adverse effect (Zariwala et al., 2012). GECIs can be targeted to the plasma membrane of astrocytes (Shigetomi et al., 2010) and their specific expression by astrocytes *in vivo* is being developed using viral constructs and Tg mouse lines (see below). Finally new GECI variants are being generated having greater signal to noise ratio, different Ca^2+^-binding affinities, and different spectral properties (Horikawa et al., 2010; Zhao et al., 2011; Ohkura et al., 2012; Akerboom et al., 2013; Chen et al., 2013b) which further enlarge their utility for studying the role of astrocytes on synaptic transmission (Tong et al., 2013).

**Figure 1:**
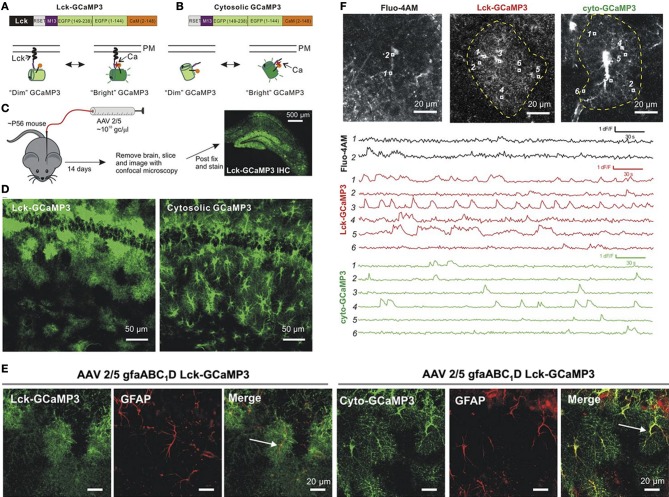
**Expression of GCAMP3 in astrocytes and comparison of Ca^2+^ signals detected with Fluo-4, cyto-GCAMP3, and Lck-GCAMP3. (A,B)** Illustration of the membrane-targeted Lck-GCAMP3 and cytosolic non-targeted Cyto-GCAMP3. **(C)** Protocol of AAV2/5 injections into a mouse hippocampus. **(D)** Confocal images in CA1 stratum radiatum for Lck-GCaMP3 and cyto-GCaMP3. **(E)** Colocalization between Lck-GCaMP3 and cyto-GCaMP3 with the astrocytic marker GFAP. **(F)** Ca^2+^ signals imaged with Fluo-4 (black traces), cyto-GCaMP3 (green traces), and Lck-GCaMP3 (red traces). Top, representative images of astrocytes loaded with Fluo-4AM, Lck-GCaMP3 or cyto-GCaMP3. ROIs are shown in each image, and their time-lapse intensities are shown below. Adapted from (Shigetomi et al., 2013b).

Among the most recent GECIs, several variants of the original GFP-based Ca^2+^ sensor GCaMP1 (Nakai et al., 2001) have been tested in astrocytes: GCaMP2 (Hoogland et al., 2009), GCaMP3 (Shigetomi et al., 2010, 2013b; Tong et al., 2013), GCaMP5 (Akerboom et al., 2012), and red GECIs (Akerboom et al., 2013), as well as Case12 (Souslova et al., 2007; Gourine et al., 2010), and yellow Cameleon YC3.60 (Atkin et al., 2009). In neurons GCaMP5G and GCaMP6 variants have been shown to produce a higher signal-to-noise ratio than GCaMP3 and can detect Ca^2+^ changes evoked by single action potential (Akerboom et al., 2012). GCaMP3, GCaMP5G, and GCaMP6 are all compatible with two-photon excitation at 910-930 nm (Akerboom et al., 2012; Mutze et al., 2012). Recently, Khakh's group (Tian et al., 2009; Shigetomi et al., 2010, 2013b) compared Ca^2+^ changes in astrocytes using a membrane-permeable Ca^2+^ indicator (Fluo4-AM) with those detected with two GECIs, the cytosolic GCaMP3 and a membrane-targeted Lck-GCaMP3 (Figure 1). Ca^2+^ signals were recorded with a confocal microscope at the surface of acute hippocampal slices from adult mice. Specific astrocytic targeting of GCaMP3 and Lck-GCaMP3 was obtained using a short version (gfaABC1D) of the human glial fibrillary acidic (hGFAP) promoter. Unlike Fluo4-AM which diffuses poorly to the thin astrocyte processes (Reeves et al., 2011), both GCaMP3 and Lck-GCaMP3 reported a wealth of Ca^2+^ signals in distant thin astrocytic processes with relatively less activity in the soma and proximal processes. Interestingly, (i) spontaneous Ca^2+^ rises recorded with the GCaMP3 are highly localized and desynchronized, as suggested previously from whole-cell dye loading single astrocytes with higher dye concentrations through the patch pipette (Nett et al., 2002; Di Castro et al., 2011; Panatier et al., 2011); (ii) spontaneous somatic activity does not appear to integrate the signals generated locally in the thin processes; (iii) using Lck-GCaMP3, a new Ca^2+^ signaling pathway has been suggested in astrocytes involving the A1 transient receptor potential (TRPA1) channel (Shigetomi et al., 2011) that has been proposed to contribute to D-serine release (Shigetomi et al., 2013a). In summary, earlier studies using membrane-permeable Ca^2+^ indicators may have underestimated the variety of Ca^2+^ signaling mechanisms, and missed local interactions between astrocytes and neurones.

GECIs have been introduced only recently compared to chemical Ca^2+^ indicators that have been used since the 80s. Therefore, their photophysical properties and impact on intracellular Ca^2+^ homeostasis have not yet been characterized to the same extent as their chemical counterparts (Perez Koldenkova and Nagai, 2013). For example, the Ca^2+^affinity of many GECIs has not been yet determined in the complex intracellular milieu; their binding kinetics (on- and off-rates for Ca^2+^ binding), as well as their aggregation and bleaching rates are not well established. Open questions concern their precise mobility, local concentration, Ca^2+^ buffer capacity and subcellular localization, which can be engineered by adding genetically encoded targeting sequences, as done with Lck-GCaMP3 (Shigetomi et al., 2010). However, the capacity of GECIs to specifically detect local Ca^2+^ signals in a population of astrocytes together with their low photobleaching and high signal-to-noise ratio seem to outweigh these limitations. Indeed experiments become feasible that were simply not possible with earlier small-molecule chemical indicators. The new Ca^2+^ data with the GECIs provide intriguing clues to explore astrocyte functions but also present new challenges. New tools will be needed to reliably detect and quantify the wealth of rapid asynchronous and local fluorescence changes. The mechanisms and functional significance of these Ca^2+^ signals are far from being understood. Given the striking difference between fluorimetric Ca^2+^ signals detected with chemical Ca^2+^ indicators and GECIs, the relation between neuronal activity and astrocytic Ca^2+^ signals needs to be re-investigated under physiological and pathological conditions using acute brain slices, and anesthetized or non-anesthetized mouse preparations.

### Optical methods for imaging astrocyte activity

Imaging GECIs with two-photon microscopy holds important potential for monitoring astrocytic Ca^2+^ signals *in situ*. However, major challenges remain when imaging astrocyte signals in the neuropil. The limited spatial resolution is an issue when it comes to tell apart morphological changes and Ca^2+^ signals. An astrocyte process occupies only a small fraction of the two-photon excitation volume and, this fraction will become smaller with increasing imaging depth, for which two-photon resolution degrades due to scattering and wave front aberrations (Chaigneau et al., 2011). Thus, subtle morphological changes expected to modulate synapse coverage and modulate astrocyte-neuron interactions are unlikely to be resolved in two-photon microscopy and will be confounded with Ca^2+^ changes.

Two-photon microscopy combined with two-photon stimulated emission depletion (STED) (Ding et al., 2009; Li et al., 2009b; Moneron and Hell, 2009; Nägerl and Bonhoeffer, 2010) increases lateral resolution and super-resolution imaging of dendritic spines in live mouse brain *in vivo* has been recently reported (Berning et al., 2012). Two-photon-STED is attractive because it brings two-photon resolution closer to typical dimensions of astrocyte processes but it aggravates the temporal resolution problem inherent to scanning microscopies where temporal resolution depends on the number of pixels (i.e., the sampling rate imposed by the spatial resolution) and the dwell time per pixel (i.e., the number of photons available). As spatial resolution is privileged in 2PE-STED, the number of image pixels increases as the resolution gain squared, and the temporal resolution drops accordingly. Simultaneous multi-spot detection (Cheng et al., 2011; Grosberg et al., 2012; Ducros et al., 2013) speeds up image acquisition by an order of magnitude compared to conventional raster scanning, but does not fundamentally address the temporal undersampling problem. Finally, STED resolution scales with the square-root of power of the depletion beam so that STED increases the already high light burden of two-photon imaging (Koester et al., 1999). Faster imaging, i.e., shorter pixel dwell times will thus require better fluorophores with higher fluorescence quantum yield, greater depletion efficiency, and higher photostability, all at the same time. Also, while these arguments already hold for imaging a single focal plane, their weight increases when it comes to imaging Ca^2+^ changes in three dimensions along a branch or an entire astrocyte. In summary, major technological advances are still needed to image small signals in fine astrocyte processes and track their subtle morphological changes.

## Activation of astrocytes

Following neuronal activity, the activation of astrocytes is mediated by neurotransmitter released from synaptic terminals (Porter and McCarthy, 1996; Wang et al., 2006). The subsequent release of gliotransmitters from mature protoplasmic astrocytes has been reported to depend upon G_*q*_ GPCR activation leading to astrocytic type-2 IP_3_ receptor (IP_3_R2) activation and Ca^2+^ release from the endoplasmic reticulum [reviewed in (Halassa et al., 2007)]. While this pathway has been implicated in gliotransmitter release, the mechanisms and the concept of gliotransmission remains debated (Agulhon et al., 2008; Fiacco et al., 2009; Hamilton and Attwell, 2010) in part because of our inability to selectively activate Ca^2+^ signals in astrocytes. The exogenous generation of Ca^2+^ signals that mimic those evoked by neuronal stimuli should clarify the interactions between neurons and astrocytes.

### Pharmacogenetics

Since most cell types in the brain express an overlapping array of GPCRs, conventional pharmacological approaches consisting of bath application or local pipette perfusion of G_*q*_ GPCR agonists to evoke Ca^2+^ elevations in astrocytes *in situ* lack cellular selectivity, For instance, one of the agonists most frequently used to stimulate astrocytes, dihydroxyphenylglycine (DHPG), a group I metabotropic receptor (mGluR) agonist, has direct effects on neurons, and elicits neuronal Ca^2+^ elevations, long-term depolarization (Mannaioni et al., 2001; Rae and Irving, 2004), and potentiation of N-methyl-D-aspartate (NMDA) receptor-mediated currents (Benquet et al., 2002). Therefore, the use of G_*q*_ GPCR agonists, not only DHPG but also many other agonists, will lead to direct activation of neuronal receptors.

To overcome these limitations, a novel Tg mouse model was created (Fiacco et al., 2007) using the G_*q*_ GPCR MrgA1 receptor normally expressed by nociceptive sensory neurons (Dong et al., 2001). In the MrgA1 mouse model: (i) the GFP-tagged MrgA1 receptor is expressed selectively by astrocytes using an inducible tet-off system transcribed from a tet (tetO) minimal promoter; (ii) it is not activated by endogenous ligands found in brain; (iii) its ligand, the FMRF peptide, does not activate any endogenous brain G_*q*_ GPCRs. These mice were crossed with mice in which the tetracycline transactivator (tTA) is targeted to astrocytes using the hGFAP promoter. In the absence of doxycycline, tTA binds to tetO and drives expression of the MrgA1-GFP construct selectively in astrocytes and the MrgA1 receptor is functional in most astrocytes (Fiacco et al., 2007).

MrgA1-receptor-mediated Ca^2+^ release from astrocytic internal Ca^2+^ stores did not affect synaptic transmission and plasticity (Fiacco et al., 2007; Agulhon et al., 2010), raising questions about the ability of astrocytes to undergo Ca^2+^-dependent gliotransmitter release. Using the same MrgA1 mouse model, it was later shown that instead, astrocytic MrgA1R-mediated Ca^2+^ elevations potentiate glutamate and K^+^ uptake (Wang et al., 2012; Devaraju et al., 2013). The MrgA1 Tg mouse model demonstrates the interest of a pharmacogenetic approach to investigate the role of astrocytic Ca^2+^ in acute slices and *in vivo*. However, astrocytes are heterogeneous (Zhang and Barres, 2010) and they are likely to exhibit different functions depending on the brain area. Consequently, continued improvements are needed to activate discrete populations of astrocytes in specific brain areas. The combination of pharmacogenetic with the use of adeno-associated viral technology to deliver the expression of genetically engineered new G_*q*_ GPCRs (Wess et al., 2013) is a promising approach (see below). Additionally, two-photon uncaging of caged FMRF at the vicinity of thin astrocyte processes should better mimic synaptically-induced G_*q*_ GPCR activation and therefore help addressing further the role of this signaling pathway in astrocyte physiology.

### Optogenetics

Over the last decade, the development of new photoswitchable genetically encoded channels and receptors to activate and inactivate specific neuronal subtypes had a significant impact on Neuroscience. The simultaneous methodological advances in several fields: (i) optics for photoactivation and imaging *in situ*, (ii) molecular engineering for developing new photoswitchable proteins, (iii) molecular biology for specific targeting of the light sensitive proteins, have been instrumental for the success of optogenetics in elucidating the function of neuronal circuits (Szobota and Isacoff, 2010; Fenno et al., 2011; Miesenbock, 2011). The most popular photoswitchable channel to activate neurons is the H314R channelrhodopsin 2 [ChR2(H314R)], a variant of the wild type ChR2 with reduced desensitization (Nagel et al., 2005). ChR2 is a cationic channel highly permeable to proton (P^+^_H_/P^+^_Na_ ~ 10^6^) but weakly permeable to Ca^2+^ (P^2+^_Ca_/P^+^_Na_ ~ 0.117) (Nagel et al., 2003; Lin et al., 2009). In neurons, its photoactivation triggers Ca^2+^ elevations which depend mainly on the secondary activation of voltage-gated Ca^2+^ channels (VGCC) (Nagel et al., 2003; Zhang and Oertner, 2007; Li et al., 2012).

**Figure 2:**
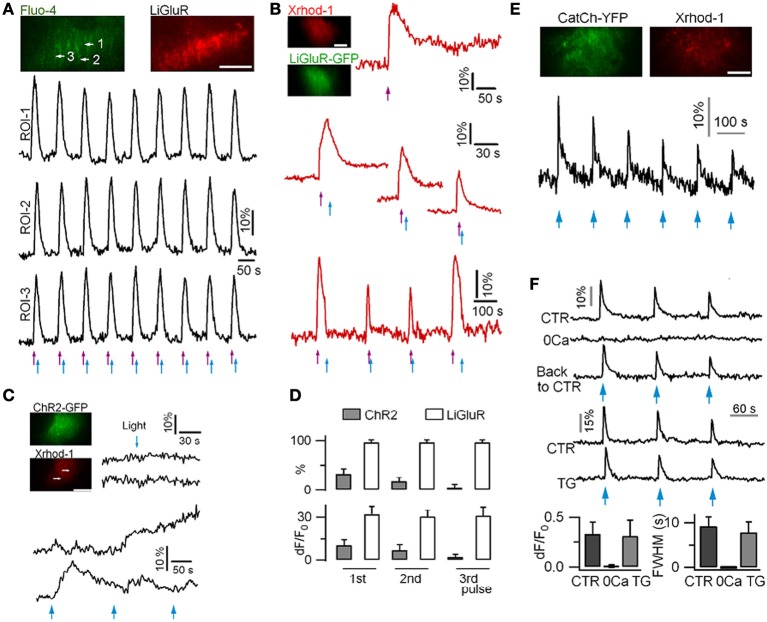
**Ca^2+^ signals recorded in mouse cortical astrocytes in culture using LiGluR, ChR2(H134R) and CatCh photoactivation. (A)** Light-gated Ca^2+^ rises in an astrocyte expressing LiGluR-mRFP. Ca^2+^ rises were imaged with dual-color TIRFM and repetitively switched on and off by 385-nm (violet arrows, 0.3 mW/mm^2^, 50 ms) and 488-nm (blue arrows, 39.1 mW/mm^2^, 200 ms) light pulses, respectively. **(B)** LiGluR(GFP)-gated astrocytic Ca^2+^ elevations monitored with the red-fluorescent Ca^2+^ dye Xrhod-1. **(C)** In astrocytes expressing ChR2(H134R), short photoactivation (458-nm, 27.3 mW/mm^2^, 500 ms) of ChR2 failed to evoke near-membrane Ca^2+^ elevation (top). Longer light pulses (458-nm, 1 s) evoked variable Ca^2+^ signals (bottom). **(D)** Comparison of the percentage of astrocytes showing light-gated Ca^2+^ rises, and of the amplitude of Ca^2+^ responses in LiGluR- and ChR2-expressing astrocytes. LiGluR evokes more reliable and reproducible Ca^2+^ rises in astrocytes. **(E)** CatCh-evoked astrocyte Ca^2+^ elevations following blue light photoactivation (1 s, 458-nm). **(F)** CatCh-induced Ca^2+^ signaling was abolished in the absence of extracellular Ca^2+^, but unaffected when ER Ca^2+^ store is perturbed by thapsigargin (TG). Bars, 10 μm. Adapted from (Li et al., 2012).

Attempts have been made to photoactivate protoplasmic astrocytes. *In situ* experiments suggest that the photoactivation of ChR2-expressing astrocytes can trigger gliotransmitter release (Gradinaru et al., 2009; Gourine et al., 2010; Sasaki et al., 2012; Chen et al., 2013a). In the rat brain stem retrotapezoid nucleus, ChR2-expressing astrocytes responded to long lasting (20–60 s) illumination by slow Ca^2+^ rises that lasted for minutes (Gourine et al., 2010). In the hippocampal CA1 region, blue light pulses induce rapid time-locked Ca^2+^ signals in astrocytes (Chen et al., 2013a). However, our own experiments using mouse cortical astrocytes in culture, show that ChR2 activation induces variable and weak Ca^2+^ elevations (Li et al., 2012). Instead we found that the activation of the Ca^2+^-permeable light-gated glutamate receptor (LiGluR) [reviewed in (Szobota and Isacoff, 2010)], and the Ca^2+^-translocating ChR2 (CatCh) (Kleinlogel et al., 2011) evokes reliable and robust Ca^2+^ signals in astrocytes (Figure 2). We attributed the low efficacy of ChR2 in astrocytes to its relatively weak Ca^2+^ permeability (Nagel et al., 2003; Lin et al., 2009), and to the absence of VGCC in protoplasmic astrocytes (Carmignoto et al., 1998; Parpura and Verkhratsky, 2012). Interestingly, LiGluR can be rapidly switched ON and OFF to mimic endogenous Ca^2+^ signals recorded with the GCaMP3 (Shigetomi et al., 2013b). Finally, LiGluR activation induces a large Ca^2+^ influx that is further shaped by internal stores, while CatCh activation generates a Ca^2+^ influx insensitive to internal Ca^2+^ store depletion, indicating that LiGluR and CatCh are interesting tools to activate differentially selective Ca^2+^ signaling pathways and to study their downstream effects.

Astrocytes express a rich repertoire of metabotropic G_q_, G_i_/o, and G_s_ GPCRs (Porter and McCarthy, 1997). New light-gated proteins that mimic these GPCR-mediated pathways have been developed (Schroder-Lang et al., 2007; Airan et al., 2009; Ryu et al., 2010; Gutierrez et al., 2011; Stierl et al., 2011; Levitz et al., 2013), but they have not yet been tested on astrocytes. Since astrocytes express store-operated Ca^2+^ channels Orai1 (Akita and Okada, 2011; Linde et al., 2011; Moreno et al., 2012), it should also be of interest to activate them with the new photosensitive synthetic protein LOVS1K that reversibly translocates to Orai1 channels and generates either local Ca^2+^ signals at the plasma membrane or global Ca^2+^ signals upon repeated photoactivation (Pham et al., 2011).

### Optical methods to photoactivate astrocytes

While imaging morphological dynamics of astrocytic fine processes may not yet be possible, stimulating astrocytes *locally* with light is more promising because the astrocyte-specific expression of light-sensitive Ca^2+^-permeable ion channels circumvents the optical resolution problem, and therefore even one-photon whole-field illumination is sufficient to stimulate specifically the astrocytes. A more specific photoactivation of a subset of cells, and the local subcellular stimulation of a single astrocyte can be achieved using spatial light modulators [reviewed in (Maurer et al., 2011)] to shape the light (Shoham, 2010; Vaziri and Emiliani, 2012; Papagiakoumou, 2013). One-photon digital holography allows photoactivation within precisely shaped regions of interest at or near the tissue surface.

Combining digital holography and two-photon excitation with temporal focusing to modulate the temporal width of the pulsed laser, several groups reported shaped two-photon excitation deep inside scattering tissue (Andrasfalvy et al., 2010; Papagiakoumou et al., 2010). The spatial patterns thus generated are robust against scattering and remained confined at depths of 100 μm (Papagiakoumou, 2013). Combining optogenetics, shaped photoactivation and two-photon imaging for the optical readout of astrocytes (combined with electrophysiology for recording neuronal signals) holds important promises for interrogating interactions between neurons and astrocytes in intact brain tissue. To probe specific signaling pathways, wave-front light shaping can be combined with uncaging of classical IP_3_ and Ca^2+^ cages (Ellis-Davies, 2011), and new endothelin cage (Bourgault et al., 2007).

## Monitoring gliotransmitter release

Among the mechanisms of gliotransmitter release, Ca^2+^-regulated exocytosis of synaptic-like small vesicles has been proposed as a major pathway (Cali et al., 2009). Total internal reflection fluorescence microscopy (TIRFM) is a powerful technique to monitor single-vesicle behavior and to study the mechanisms of vesicular docking and fusion in cultured cells (Holz and Axelrod, 2008). Since cultured astrocytes may differ from their *in situ* counterparts, the physiological relevance of the findings made in culture with TIRFM need to be validated *in situ* using other approaches.

TIRFM has been used to visualize near membrane single vesicles and monitor single vesicle fusion in cultured astrocytes (Bezzi et al., 2004; Zhang et al., 2004; Bowser and Khakh, 2007; Li et al., 2008; Malarkey and Parpura, 2011; Potokar et al., 2013). In early experiments (Bezzi et al., 2004), the fluorescent weak base acridine orange (AO) was used to report exocytosis, and the vesicular glutamate transporter (VGLUT) tagged with the enhanced GFP (EGFP) was overexpressed to identify the AO-positive vesicles. Following DHPG application, rapid millisecond Ca^2+^-dependent flashes of AO-labeled vesicles were detected and interpreted as the exocytosis of glutamatergic vesicles (Bezzi et al., 2004; Domercq et al., 2006). This interpretation was soon complicated by studies showing that AO metachromasy results in its simultaneous emission of green and red fluorescence, which invalidates the identification of the AO-positive vesicles with EGFP labeling (Nadrigny et al., 2006, 2007). It has also been shown that the flash events of AO-loaded astrocyte vesicles are not solely due to exocytosis but also reflect intracellular vesicle photolysis (Jaiswal et al., 2007; Li et al., 2008), due to the action of AO as a photosensitizer. Styryl pyridinium FM dyes, established markers of vesicular release in neurons (Rizzoli and Betz, 2005), were also used to label the astrocytic vesicular compartments and report exocytosis. However, FM dyes are handled differently by neurons and astrocytes (Li et al., 2009a) and they label mainly lysosomes (Zhang et al., 2007; Li et al., 2008; Liu et al., 2011).

Later, the genetically encoded exocytotic reporter, pHluorin, has emerged as a valuable tool to monitor astrocyte vesicle exocytosis. As a pH-sensitive GFP mutant, pHluorin fluorescence is quenched in the acidic vesicle lumen and becomes bright upon vesicle fusion when the fluorescent protein is exposed to external neutral pH (Miesenbock et al., 1998). Since there was evidence that astrocytes release glutamate via Ca^2+^-regulated exocytosis (Cali et al., 2009), pHluorin was targeted to the lumen of putative glutamatergic vesicles in astrocytes by using the fusion protein VGLUT1-pHluorin (Marchaland et al., 2008). TIRFM imaging of single vesicles in cultured astrocytes labeled with VGLUT1-pHluorin revealed fusion events occurring within hundreds of milliseconds after Ca^2+^ rise evoked by either mGluR (Marchaland et al., 2008) or purinergic P2Y1 receptor activation (Santello et al., 2011). These results were consistent with those obtained by the same lab using AO-labeled (Bezzi et al., 2004; Domercq et al., 2006), and FM-labelled astrocytes (Cali et al., 2008).

Different kinetics have been reported for the exocytosis of the putative glutamatergic vesicles in astrocytes when using another pHluorin-based exocytotic reporter synaptopHluorin (spH), a chimeric construct tagging the luminal side of synaptobrevin 2 (Burrone et al., 2006). As synaptobrevin 2 appears to colocalize with VGLUT1 on the same vesicles in astrocytes (Montana et al., 2004; Zhang et al., 2004; Liu et al., 2011), expressing spH in astrocytes leads to the labeling of VGLUT1-positive vesicles (Bowser and Khakh, 2007; Liu et al., 2011). However, unlike VGLUT1-phluorin that reports fast millisecond kinetics of exocytosis (Cali et al., 2008; Santello et al., 2011), spH-labeled vesicles undergo slow exocytosis that is loosely coupled to stimulation, with most events occurring ~2 min after P2 receptor-mediated Ca^2+^ rise (Malarkey and Parpura, 2011), and within hundreds of milliseconds following Ca^2+^ increase evoked by mechanical stimulation (Liu et al., 2011; Malarkey and Parpura, 2011).

Genetically encoded reporters of exocytosis set the stage for investigating the mechanisms of astrocyte exocytosis and for addressing several remaining questions. First, the reasons for the variable fusion kinetics of putative VGLUT-positive vesicles remain to be elucidated. Second, several studies failed to detect the presence of VGLUT expression in astrocytes (Cahoy et al., 2008; Juge et al., 2010; Li et al., 2013), therefore, new experiments are needed to clarify the molecular identity of the VGLUT-positive vesicles. Recently, a new genetically encoded red pH-sensitive probe, pHTomato, has been introduced to image single vesicle exocytosis (Li and Tsien, 2012). It should allow monitoring exocytosis, and, simultaneously, activating Ca^2+^ signal with optogenetic tools that typically require blue light illumination (Li et al., 2012). Finally, the new genetically encoded glutamate sensor, iGluSnFR, shows fast kinetics (Marvin et al., 2013) and is potentially suitable for fast real-time recording of glutamate release from astrocytes. Combining it with TIRFM detection of single vesicle exocytosis would help to clarify the relative contribution of vesicular vs. non-vesicular release pathways to glutamate release (Kimelberg et al., 2006; Li et al., 2012; Woo et al., 2012). Fourth, a combination of fast two-photon imaging, local photoactivation and genetically targeted expression of pHluorins in slice and *in vivo* must validate earlier findings from cell-culture studies.

## Targeting genetically encoded proteins to astrocytes

Most optical techniques available today lack the spatial resolution for imaging thin astrocyte processes. Bulk-loaded Ca^2+^ indicators that label both astrocytes and neurons, report mixed signals. However, using GECIs targeted selectively to astrocytes, it becomes possible to record astrocyte-specific Ca^2+^ signals with standard imaging techniques, like confocal, spinning-disk confocal, and two-photon microscopies. Similarly by targeting the light-gated proteins, selective photoactivation of astrocytes can be achieved (see below). Therefore, targeting the GECIs and the light-gated channels/receptors to astrocytes *in situ* is a critical step, and several strategies have been used. First, plasmids can be electroporated *in utero* (Yoshida et al., 2010) but the yield of electroporation is variable, therefore, protocols using intracerebral and intravenous injections of viral constructs and Tg mouse lines are being favored.

Viral constructs are relatively easy to generate and adeno-associated virus (AAV) are the most widely used for astrocyte infection (Table 1). Intracerebral injections of lentivirus (LV) and adenovirus (AV) (Liu et al., 2008; Colin et al., 2009; Gourine et al., 2010) have been limited due to their possible toxic effects. Among the AAV variants, the serotype 5 with a high tropism for astrocytes (Ortinski et al., 2010), is commonly used for packaging the DNA constructs. Following intracerebral viral injections, the area of infection extends well beyond the injection site and the expression of the reporter genes is stable for months. In adult mice, intravenous injections of AAV serotype 9 labels mostly astrocytes rather than neurons (Foust et al., 2009). Intraventricular injection of AAV serotype 8 at postnatal day 3 (P3) labels preferentially astrocytes (Kim et al., 2013). New serotypes have been developed using a directed evolution approach with higher transduction level for astrocytes leading to 94% specific expression in retinal Müller glial cells after intravitreal injection (Klimczak et al., 2009). This seems to be promising to reduce the virus titer needed for expression. More specific targeting can be achieved by inserting an astrocyte-specific promoter. The 2.2 kb hGFAP promoter targets selectively ChR2 to astrocytes using LV constructs (Gradinaru et al., 2009). However, since AAVs cannot carry constructs larger than 4.7 kb, a shorter 681 bp hGFAP promoter (gfaABC_1_D also called gfa104) (Lee et al., 2008) has been used to target selectively EGFP, ChR2, GECIs (case12, GCaMP3, Lck-GCaMP3), and a pleckstrin homology (PH) domain of phospholipase C-like protein p130 (p130PH) to astrocytes (Ortinski et al., 2010; Xie et al., 2010; Shigetomi et al., 2013b).

**Table 1:** **Viral constructs for specific targeting mouse astrocytes**.

**Viral construct^a^**	**Applications^b^**	**Brain regions**	**Specificity**	**References**
AAV1/2-GFAP-GFP AAV1/2-CBA-GFP^c^	IC at P0, P90	Cortical and subcortical	AAV1/2-GFAP-GFP expressed mostly ALDH1L1(+) cells, AAV1/2-CBA-GFP specific for subcortical neurons at P90	von Jonquieres et al., 2013
AAV2/5-gfaABC_1_D-Lck-GCaMP3^d^ AAV2/5-gfaABC_1_D-GCaMP3	IC at P49 to P63	CA1 region	Labels GFAP(+) cells	Shigetomi et al., 2013b
AAV8-CBA^e^-eYFP-2A-tTA(2S)	IVE at P3	Whole brain	Labels S100β (+) cells	Kim et al., 2013
AAV2/5-, AAV2/8-, AAV2/9-CBA-EGFP	IVE at P0 to P3	Whole brain	AAV2/5 at P0 to P3 transduces mostly GFAP(+) cells, AAV2/8, AAV2/9 at P3 transduce mostly GFAP(+) cells	Chakrabarty et al., 2013
AV-CMV-Flox-ChR2-mCherry (injected in hGFAP-Cre line)	IC at P14	CA1 region	Labels GFAP(+) cells, but not NeuN(+) cells	Chen et al., 2013a
AAV1-, AAV8-, AAV9, AAVrh10-CAG-GFP	IV at P0, P5, P14, P42	Whole brain	Both neurons and astrocytes were labeled	Miyake et al., 2011
scAAV7-, AAV9-, rh10-rh39-, rh43-CB-EGFP^f^	IV at P1	Whole brain	Both neurons and astrocytes were labeled	Zhang et al., 2011
AV-mCMV-gfaABC_1_D-Case12 AV-mCMV-gfaABC_1_D-ChR2(H134R)-Katushka1.3	IC	Hypoglossal motor nucleus	All labeled cells are GFAP(+)	Gourine et al., 2010
AAV2/5-, AAV2/9-CMV-EGFP AAV2/5-gfa104-EGFP	IC	CA1 region	AAV5-CMV shows tropism for astrocytes	Ortinski et al., 2010
			AAV5-gfa104 >99% selectivity for astrocytes	
AAV2/5-gfaABC_1_D-mRFP-p130PH	IC	Cortex Hippocampus	Selective expression in GFAP(+) cells	Xie et al., 2010
LV-GFAP-hChR2(H134R)-mCherry	IC	Subthalamic nucleus	Labelled cells are GFAP(+)	Gradinaru et al., 2009
LV-PGK-nlsLacZ -miR124T/ LV-PGK-GLAST-miR124T Mokola pseudotyped	IC	Hippocampus, Cerbellum, Striatum	6% neuronal expression in striatum	Colin et al., 2009
scAAV9-CB-GFP	IV, tail injection at P70	Whole brain	Labels mostly astrocytes	Foust et al., 2009
LV-mCMV-gfaABC_1_D-EGFP	IC	Hypoglossal motor nucleus	All labeled cells are GFAP positive	Liu et al., 2008

a We keep the names given in the quoted papers. Notation AAV2/x (also called AAVx) stands for pseudotyped AAV. ITR of coding plasmid derived from AAV serotype 2 and capsid sequence from serotype x.

b IC, intracerebral; IV, intravenous; IVE, intraventricular; P0 day of birth.

c AAV1/2 chimeric AAV with capsid proteins from AAV1 and AAV2.

d gfaABC_1_D (also called gfa104) is a short (681 bp) version of the hGFAP promoter.

e CBA, CB, CAG, hybrid promoter composed of CMV early enhancer element and chicken β-actin promoter.

f Sc, self complementary, see

Gene targeting of viral constructs can also be achieved by the Cre-Lox or tetO-tTA strategies injecting floxed (or flexed) viral constructs in Cre mouse lines, or tetO virus in the tTA mouse lines (Pfrieger and Slezak, 2012). Several astrocyte-specific Cre- and tTA-expressing mouse lines have been generated (Table 2). When a Cre-dependent ChR2-expressing virus was injected in hippocampus (Chen et al., 2013a) of a P14 hGFAP mouse line (Casper and McCarthy, 2006), selective expression of ChR2 was obtained in the astrocytes.

**Table 2:** **Transgenic (Tg) mouse lines for specifically targeting mouse astrocytes**.

**Tg mouse lines**	**Brain regions studied**	**Specificity**	**References**
Mlc1-tTA & TetO-ChR2(C128S)-YFP	Cerebellum	Light-evoked current in the Bergmann glia	Sasaki et al., 2012; Tanaka et al., 2012
hGFAP-CreER^T2^ & Mecp2^+/Stop^	Whole brain	<5% expression in neurons	Lioy et al., 2011
hGFAP-tTA & tetO-MrgA1-GFP	Hippocampus	Labels GFAP(+) cells	Fiacco et al., 2007; Agulhon et al., 2010
S100β-YC3.60 cameleon	Whole brain	Labels astrocytes, NG2 cells and oligodendrocytes	Atkin et al., 2009
S100β-Cre & Cx43(fl/fl)	Cerebellum	Specific Cx43 deletion in Bergmann glia and molecular layer astrocytes	Tanaka et al., 2008
hGFAP-CreER^T2^ hGFAP-MerCreMer	Hippocampus Cortex Cerebellum	Specific recombination in cortical astrocytes and Bergmann glia in hGFAP-CreER^T2^ and hGFAP-MerCreMer lines	Casper et al., 2007
GLAST-CreER^T2^ Cx30-CreER^T2^ ApoE-CreER^T2^ AQ4-CreER^T2^	Whole brain	Specific labeling of GFAP- and S100β-positive astrocytes in cortex, hippocampal CA1 region and cerebellum in Cx30-CreER^T2^ and GLAST-CreER^T2^ lines. Density of labeled astrocytes is higher in Cx30-CreER^T2^ line. ApoE-CreER^T2^ and AQ4-CreER^T2^ lines do not label specifically the astrocytes.	Slezak et al., 2007
GLAST-CreER^T2^	Cortex Hippocampus Cerebellum	Tamoxifen injection in adult mice induce specific labeling of S100β-positive astrocytes in cortex; olfactory bulb, dentate gyrus and cerebellum. Adult-born doublecortin-positive neurons were labeled. Tamoxifen injection at E18 induces recombination both in neurons and astrocytes	Mori et al., 2006
human or mouse GFAP-Cre	Whole brain	Recombination occurs both in astrocytes and neurons	Casper and McCarthy, 2006
hGFAP-CreER^T2^	Whole brain	Recombination is specific in GFAP- and S100β-positive astrocytes in hippocampus and cerebellum. Recombination is weak in cortex.	Hirrlinger et al., 2006

**Figure 3:**
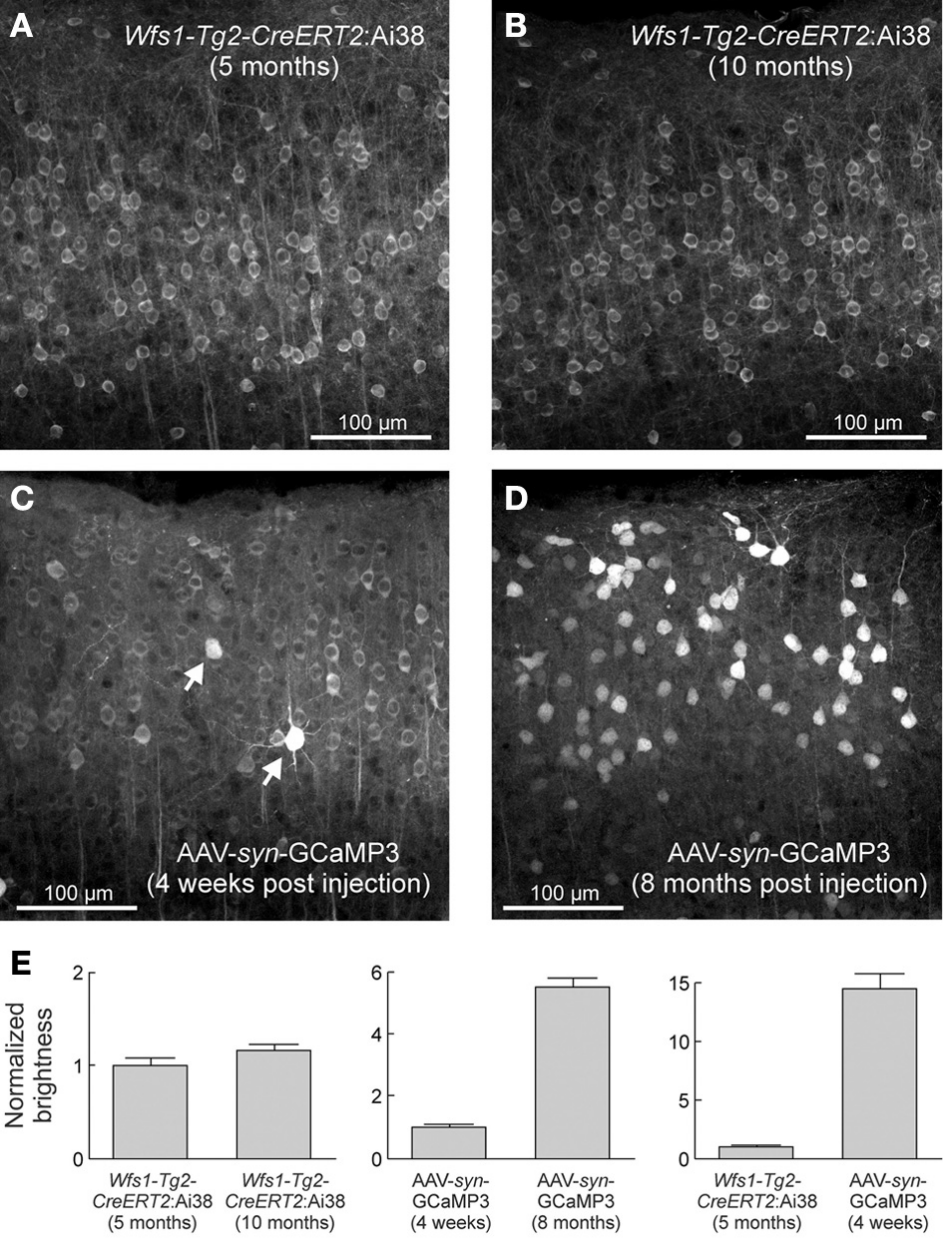
**Stable expression levels in the Ai38 ROSA26-GCaMP3 mouse line over months.** Native GCaMP3 fluorescence in layer2/3 excitatory neurons of visual cortex from Wfs1-Tg2-CreERT2:Ai38 mice **(A,B)** and adult wild-type mice injected with AAV-syn-GCaMP3 **(C,D)**. **(E)** Quantification of neuronal brightness. Error bars correspond to SEM. From (Zariwala et al., 2012), with permission.

Yet an important limitation of the viral delivery strategies needs to be taken into account. Following intracerebral injection of AAV2/5-gfa104-EGFP construct, a significant dose-dependent reactive gliosis has been observed (Reimsnider et al., 2007; Klein et al., 2008; Ortinski et al., 2010). Since gliosis is associated with changes of several signaling pathways in astrocytes (Hamby et al., 2012), it will be important to develop alternative approaches to study the role of astrocytes in physiological conditions. Introducing a sequence encoding the VIVIT peptide that interferes with the calcineurin/nuclear factor of activated T-cells signaling pathway and down regulates GFAP overexpression (Furman et al., 2012), may reduce AAV-induced gliosis. Replacing viral constructs by Tg floxed/tetO mouse lines is a very promising approach to control gliosis. Several floxed (Slezak et al., 2012; Zariwala et al., 2012) and tetO (Fiacco et al., 2007; Agulhon et al., 2010) mouse lines of interest have been generated. With a hGFAP-CreER^T2^ mouse line in which the recombination can be induced in juvenile or adult mice by tamoxifen injections, astrocyte-specific targeting has been obtained in cortex, hippocampal CA1 region, cerebellum, diencephalon and brain stem, with weaker levels of recombination in cortex (Hirrlinger et al., 2006; Lioy et al., 2011). In glutamate-aspartate transporter (GLAST)- and connexin 30 (Cx30)-CreER^*T*2^ mouse lines, astrocyte-specific recombination occurs in cortex, hippocampal CA1 region, and cerebellum. But in GLAST-CreER^T2^ and hGFAP-CreER^T2^ mouse lines, the recombination is not astrocyte-specific in brain regions including the olfactory bulb and hippocampal dentate gyrus, where neurons are also labeled (Mori et al., 2006; Slezak et al., 2007). The floxed/tetO strategy is advantageous since it does not require surgery for viral injections. A Ai38 floxed GCaMP3 reporter mouse line was generated by a knockin strategy to insert GCaMP3 into the ROSA26 locus (Zariwala et al., 2012). When the Ai38 mouse was crossed with an inducible Wfs1-Tg2-CreER^T2^ mouse line, a uniform expression of the reporter genes was obtained in cortical excitatory neurons without over expression of GCaMP3 in the nucleus as observed after cortical injection of an AAV-syn-GCaMP3 construct (Figure 3). Importantly, it appears that various Cre- and tTA-dependent mouse lines differ in their ability to induce recombination, and also the specificity of recombination can vary with the brain region, and with the age. Therefore, in order to ascertain specificity of astrocytic signal measurement and photoactivation, it will be critical to carefully validate astrocyte-specific expression, for example, by using cell type-specific antibodies and confocal microscopy.

## Perspectives

In conclusion, new genetically targeted optical and pharmacological tools allow the selective measurement and activation of astrocytic Ca^2+^ signals. These tools should be of value for studying the mechanisms of gliotransmitter release, the role of astrocytes, and more specifically the bidirectional communication between astrocytes and neurons.

### Conflict of interest statement

The authors declare that the research was conducted in the absence of any commercial or financial relationships that could be construed as a potential conflict of interest.

## Abbreviations

AAV: adeno-associated virus
ACR: astrocyte Cre reporter
ALDH1L1: aldehyde dehydrogenase 1 L1
AM: acetoxymethyl
AV: adenovirus
CatCh: calcium-translocating channelrhodopsin 2
ChR: channelrhodopsin
CMV: cytomegalovirus
Cx30: connexin 30
DHPG: dihydroxyphenylglycine
DsRed: discosoma red protein
EGFP: enhanced green fluorescent protein
GCaMP: green fluorescent protein-based Ca^2+^ sensor
GECI: genetically encoded calcium indicator
GFAP: glial fibrillary acidic protein
GFP: green fluorescent protein
GLAST: glutamate-aspartate transporter
GLT-1: glutamate transporter 1
hGFAP: human glial fibrillary acidic protein
IP_3_: inositol triphosphate
LiGluR: light-gated glutamate receptor
LimGluR: light-gated metabotropic glutamate receptor
LV: lentivirus
mGluR: metabotropic glutamate receptor
Mlc1: megalencephalic leukoencephalopathy with subcortical cysts 1
MrgA1: Mas-related gene A1
OGB1: oregon green BAPTA1
STED: stimulated emission depletion
TIRF(M): total internal reflection fluorescence (microscopy)
TA: tetracycline transactivator
TetO: tetracycline operator
Tg: transgenic
YFP: yellow fluorescent protein
